# The Antimelanogenic Effect of Inularin Isolated from Flowers of *Inula britannica* on B16F10 Melanoma Cells and Zebrafish Embryos

**DOI:** 10.4014/jmb.2003.03025

**Published:** 2020-04-02

**Authors:** Dae Kil Jang, Seung-Hyun Jung, Ji Hye Jeong, Hee Min Yoo, Ik Soo Lee, Han-Seung Shin

**Affiliations:** 1 Department of Food Science and Biotechnology, Dongguk University, Seoul 0326, Republic of Korea; 2 StarlingForce Co., Ltd., Seoul 08511, Republic of Korea; 3 Department of Applied Marine Bioresource Science, National Marine Biodiversity Institute of Korea, Seocheon 662, Republic of Korea; 4 Center for Bioanalysis, Korea Research Institute of Standards and Science, Daejeon 3113, Republic of Korea; 5 Herbal Medicine Research Division, Korea Institute of Oriental Medicine, Daejeon 3404, Republic of Korea

**Keywords:** *Inula britannica*, inularin, melanogenesis, B16F10 melanoma cells, zebrafish embryos

## Abstract

In the search for novel, natural melanogenesis inhibitors, a new sesquiterpene, inularin, was isolated from the flowers of *Inula britannica*, and the structure was determined using spectroscopic and chemical methods. The antimelanogenic effects of inularin on B16F10 melanoma cells and zebrafish embryos were evaluated. Inularin dose-dependently reduced melanocyte-stimulating hormone-induced melanin production and L-DOPA oxidation in B16F10 cells. Zebrafish embryos were used to confirm the antimelanogenic activity. Inularin significantly decreased the pigmentation of embryos compared with untreated controls.

Melanogenesis is a complex process by which melanin is produced by melanocytes in melanosomes [1]. Melanin is responsible for skin, hair, and eye color and plays a critical role in protecting the skin against ultraviolet radiation [2]. However, excessive melanin synthesis and accumulation are associated with hyperpigmentation disorders including freckles, melasma, and senile lentigo [3]. Regulation of melanin production is therapeutically important when treating pigmentation-related disorders. Several synthetic antimelanogenic agents are known; however, only a few are used as skin-whitening agents because of various safety issues [4]. There is an increasing need for natural melanogenesis inhibitors with fewer side effects, for cosmetic and medicinal applications [5].

*Inula britannica* Linnaeus (Asteraceae) is well known in China (“Xuan-Fu-Hua”), as its flowers are used in traditional Chinese medicine to treat digestive disorders, bronchitis, and inflammation [6]. In a preliminary screen using B16F10 melanoma cells, we found that a flower extract of *I. britannica* significantly reduced melanin production [7]. However, the active principle remained unclear. Further study of *I. britannica* flowers yielded a new sesquiterpene, inularin. Here, we describe its isolation and structural characterization, its antimelanogenic effect in B16F10 cells, and its effect on melanin production by embryonic zebrafish.

An ethanol extract of *I. britannica* flowers was subjected to silica gel column chromatography and divided into four fractions (A–D) based on thin-layer chromatography (TLC) data. Fraction C, which significantly reduced melanin production (65% inhibition at 50 μg/ml) by B16F10 cells, was subjected to a further series of chromatographic separation steps guided by antimelanogenic activity, leading to isolation of inularin (Fig. 1A). The material was an amorphous white powder with a molecular ion peak at *m/z* 349.1626 [M + Na]^+^ on high-resolution electron ionization mass spectrometry (HRESIMS), corresponding to a molecular formula of C_17_H_26_O_6_. The ^1^H-NMR spectrum displayed the characteristic signals of paired olefinic protons at δ_H_ 6.28 (1H, s) and 5.68 (1H, s), two oxygenated methine protons at δ_H_ 5.29 (1H, m) and 3.28 (1H, dd, *J* = 10.4, 4.8 Hz), two singlet methyls at δ_H_ 1.17 (3H, s) and 1.03 (3H, s), and an acetoxymethyl at δ_H_ 1.95 (3H, s) (Table 1). The ^13^C-NMR spectrum (Table 1), combined with the distortionless enhancement by polarization transfer (DEPT) data, yielded 17 carbon signals consisting of three methyls, five methylenes, four methines, three quaternary carbons, and two carbonyl carbons; the signals at δ_C_ 172.2 and 21.2 were assigned to an acetoxy group. These spectroscopic data suggest that inularin is a eudesmane-type sesquiterpene with an acetoxy moiety, as are its analogs [8]. A complete assignment of the chemical shifts of inularin and its substitution pattern was made using various 2D-NMR techniques. The ^1^H-^1^H correlation spectroscopy (COSY) correlations of inularin established two segments: CH(1)-CH_2_(2)-CH_2_(3) and CH(5)-CH_2_(6)-CH(7)-CH(8)-CH_2_(9) (Fig. 1B). Two oxygenated methine protons at δ_H_ 5.29 and 3.28 were assigned to H-8 and H-1 by the heteronuclear multiple bond correlation (HMBC) correlations of δ_H_ 5.29 with C-7 (δ_C_ 44.2), C-9 (δ_C_ 45.5), and C-10 (δ_C_ 40.4) and those of δ_H_ 3.28 with C-2 (δ_C_ 29.0), C-3 (δ_C_ 41.9), and C-10 (δ_C_ 40.4) (Fig. 1B). The HMBC correlations of δ_H_ 1.17 (H3-15) with C-4 (δ_C_ 72.3) and of δ_H_ 5.29 (H-8) with the acetoxy carbon (δ_C_ 172.2) indicated that the other hydroxy group was attached to C-4, and an acetoxy moiety was linked to C-8, respectively. The presence of an α-methylene-carboxylic acid was inferred by carbon signals at δ_C_ 170.0 (COOH, C-12) and 143.2 and 126.1 (C=CH_2_, C-11 and C-13) and the HMBC correlation between paired olefinic protons at δ_H_ 6.28 (H-13a) and 5.68 (H-13b) and the carboxylic carbon at δ_C_ 170.0 (C-12). Attachment of an α-methylene-carboxylic acid moiety to C-7 was indicated by the HMBC cross-peaks of H-7 with C-11 and C-12, and H-13 with C-7. The relative configuration of inularin was determined based on nuclear Overhauser effect spectroscopy (NOESY) data and comparisons of coupling constants with data from the literature. The coupling constant of H-5 (*J* = 12.0, 5.6 Hz) suggested a trans-fused eudesmane skeleton [9]. The NOE correlations (Fig. 1C) between H-1/H-5 and H3-14/H3-15 suggested that the C-1 and C-4 hydroxy groups were in the β and α configurations, respectively. The orientation of the C-7 α-methylene-carboxylic acid was β, as revealed by the NOE correlation between H-5/H-7. The coupling constants of H-9α (*J* = 14.4, 3.2 Hz) and H-9β (*J* = 14.4, 3.1 Hz) indicated that H-8 placed in ring B in an equatorial geometry, indicating a β-configuration of the acetoxy group at C-8. The absolute configuration of inularin was considered to be *1R, 4R, 5R, 7R, 8R, 10R*, as established for the co-occurring analogue eudesmolides of known absolute stereostructure [8]. Finally, the structure of inularin was established as (*1R, 4R, 5R, 7R, 8R, 10R*)-1β,4α-dihydroxy-8β-acetoxy-5α*H*-eudesma-11(13)-en-12-oic acid.

We used B16F10 cells to investigate the effect of inularin on melanogenesis. The MTT (cell viability) assay showed that inularin did not affect viability at concentrations up to 100 μM (data not shown). B16F10 cells were treated with inularin at concentrations of 10, 50, and 100 μM. As shown in Fig. 2A, the cellular melanin content was markedly increased in the melanocyte-stimulating hormone (α-MSH)-treated control group, compared with the untreated control group, and inularin significantly reduced this increase in a dose-dependent manner. Inularin at 100 μM decreased melanin production induced by α-MSH by approximately 44%, compared with the α-MSH-treated control group, whereas kojic acid exhibited only 36% inhibition even at 200 μM. As melanin synthesis is regulated by tyrosinase [10], we further examined the effect of inularin on cellular tyrosinase activity, as reflected by L-DOPA oxidation. As shown in Fig. 2B, inularin decreased the cellular tyrosinase activity of α-MSH-stimulated B16F10 cells in a dose-dependent manner.

The zebrafish serves as a useful model for phenotype-based screening of melanogenic inhibitors or stimulators [11]. We explored the effects of inularin on melanin pigmentation in embryonic zebrafish. Embryos were exposed to inularin at 10, 50, and 100 μM commencing at 10 h post fertilization (hpf), and melanin levels were assayed at 48 hpf. Melanin production was strongly increased in the untreated control group; inularin reduced pigment production in a dose-dependent manner (Fig. 3A). Inularin at 10, 50, and 100 μM reduced pigmentation by approximately 20%, 25%, and 44%, respectively, compared with the untreated control group (Fig. 3B). Phenylthiourea (PTU) reduced pigmentation by 81% at 200 μM, whereas kojic acid did not show any significant effect at 200 μM (data not shown), but reduced pigmentation by 13% at 1 mM (Fig. 3B).

Sesquiterpenes are the 15-carbon subgroup of terpenoids and characteristic of *Inula* species. In the *Inula* species, most sesquiterpenes occur in a lactonized form and exert various biological properties, including anti-inflammatory, antibacterial, anticancer, and cytotoxic activities [12, 13]. It has been reported that the cytotoxicity of sesquiterpene lactones is critically dependent upon the presence of the α-methylene-γ-lactone moiety [14, 15]. Thus, the low cytotoxicity of inularin on B16F10 cells could be related to the absence of this moiety. So far, two sesquiterpenes, 1-*O*-acetylbritannilactone and britannilactone, isolated from *I. britannica* flowers have been reported to exhibit antimelanogenic activities in vitro [7]. These results suggest that the plant antimelanogenic activity is attributable in part to its sesquiterpenes. *I. britannica* flowers and inularin are promising therapeutics for melanogenesis-related diseases. The specific mechanisms involved require further study.

## Figures and Tables

**Fig. 1:**
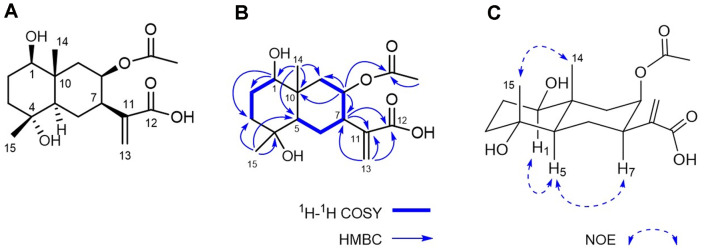
Structure of inularin isolated from the flowers of *I. britannica*. (**A**) Chemical structure of inularin. (**B**) Key ^1^H-^1^H COSY and HMBC correlations. (**C**) Key NOE correlations.

**Fig. 2:**
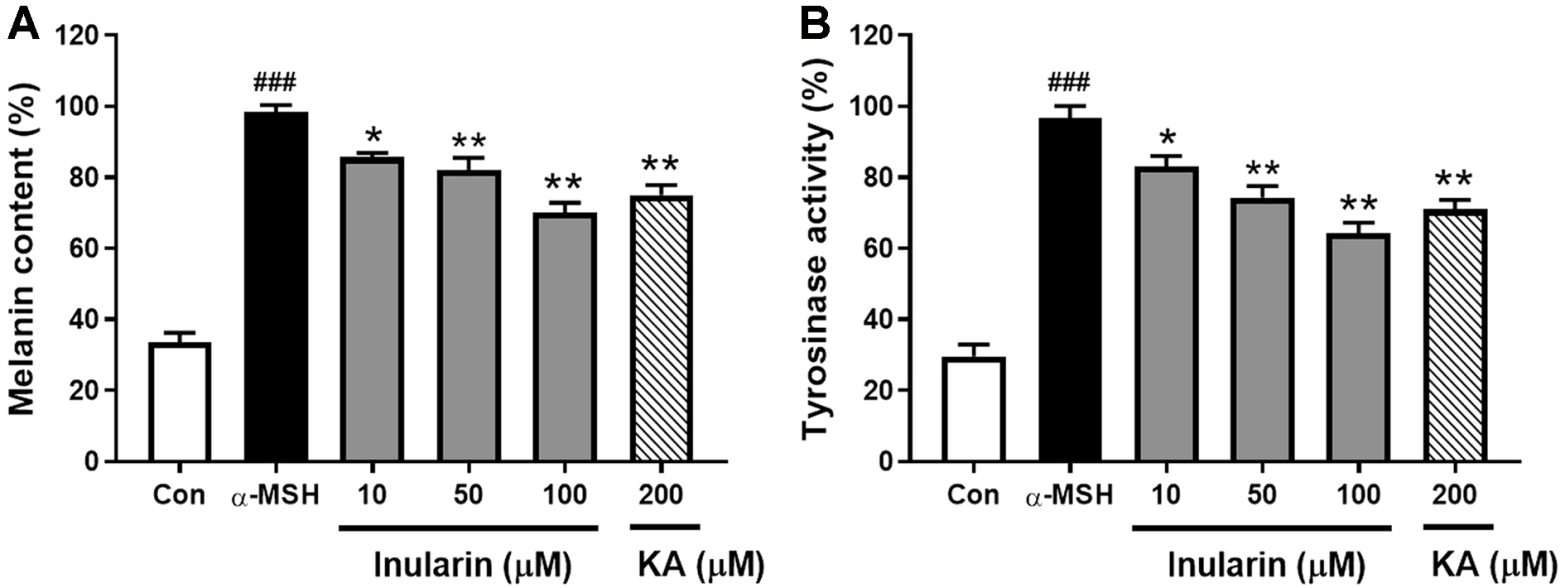
Effect of inularin on the melanogenesis of α-MSH-induced B16F10 cells. B16F10 cells were stimulated with 100 nM α-MSH for 36 h after pretreatment with the indicated concentrations of inularin (10, 50, or 100 μM) or kojic acid (KA, 200 μM) for 12 h. (**A**) The melanin content was measured at 405 nm using a microplate reader. (**B**) Tyrosinase levels were assayed by measuring L-DOPA oxidation at 475 nm using a microplate reader. The values are means ± SD from three independent experiments. ###*p* < 0.001 vs. Control, *p < 0.05 vs. α-MSH group, **p < 0.01 vs. α-MSH group.

**Fig. 3:**
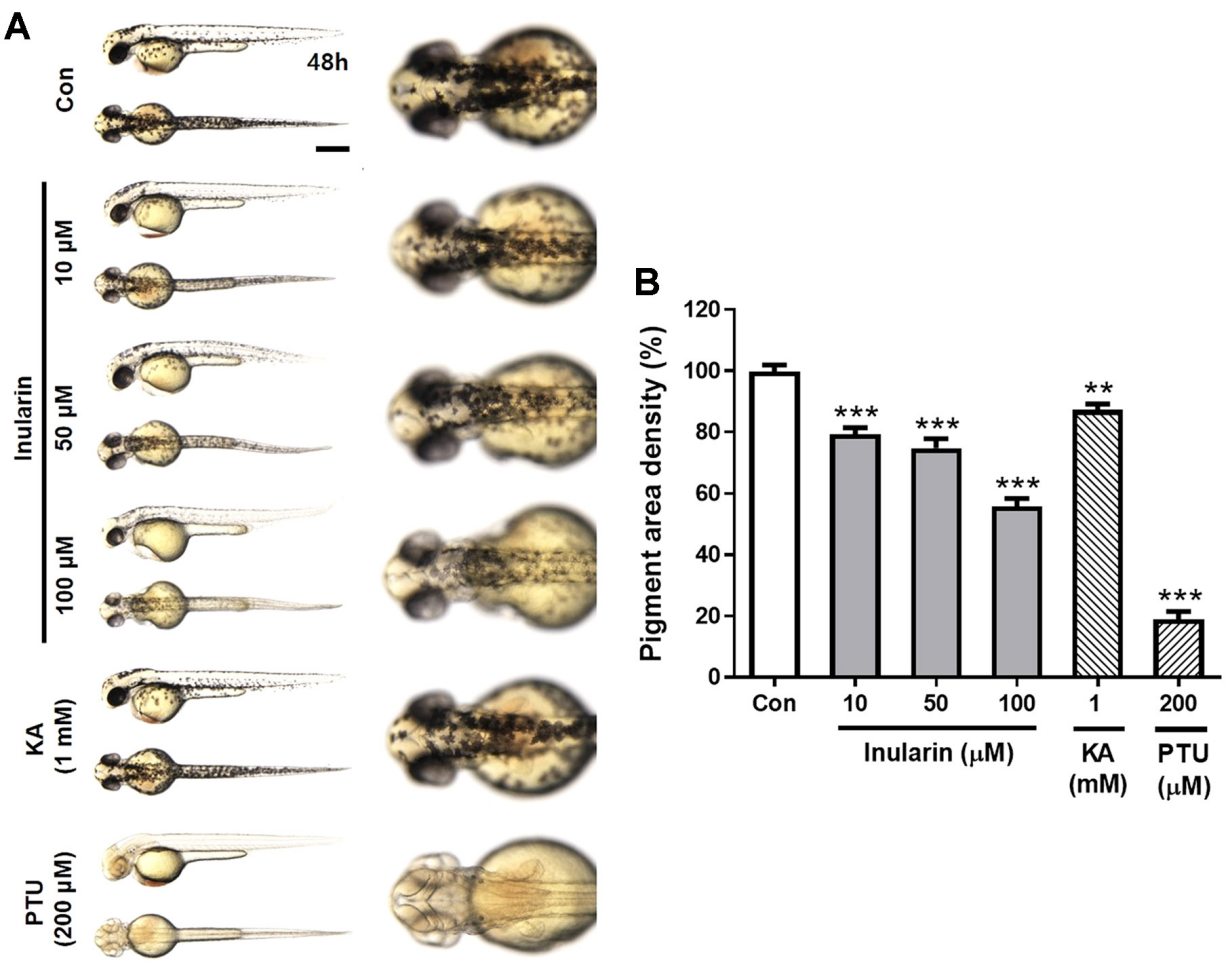
Effect of inularin on melanin production in zebrafish embryos. Zebrafish embryos were treated with inularin (10, 50, or 100 μM), kojic acid (KA, 1 mM), phenylthiourea (PTU, 200 μM), or 0.1% (v/v) DMSO (Control). (**A**) Pigmentation in the embryos was observed under a stereomicroscope (lateral and dorsal views) at 48 hpf. Scale bar: 0.5 mm. (**B**) The pigmented area density was normalized to that of control embryos using Image J software (*n* = 8). The values are means ± SEM from three independent experiments. **p < 0.01 vs. Control, ***p < 0.001 vs. Control.

**Table 1:** ^1^H- (400 MHz) and ^13^C-NMR (100 MHz) data for inularin (in MeOD).

C	δ_c_	δ_H_ (*J* in Hz)
1	80.6	3.28 dd (10.4, 4.8)
2	29.0	α: 1.69 m, β: 1.64 m
3	41.9	α: 1.55 m, β: 1.76 m
4	72.3	
5	54.6	1.45 dd (12.0, 5.6)
6	21.7	α: 1.80, β: 1.76 m
7	44.2	2.81 m
8	71.5	5.29 m
9	45.5	α: 2.17 dd (14.4, 3.2), β: 1.43 dd (14.4, 3.1)
10	40.4	
11	143.2	
12	170.0	
13	126.1	6.28 s, 5.68 s
14	16.1	1.03 s
15	22.8	1.17 s
CH_3_CO	172.2	
CH_3_CO	21.2	1.95 s
